# Late preterm antenatal corticosteroids in singleton and twin gestations: a retrospective cohort study

**DOI:** 10.1186/s12884-022-05262-1

**Published:** 2022-12-05

**Authors:** Luciana A. Vieira, Yu-Han Kao, Despina S. Tsevdos, Yan Kwan Lau, Zichen Wang, Shilong Li, Amanda B. Zheutlin, Susan J. Gross, Joanne L. Stone, Siobhan M. Dolan, Eric E. Schadt, Li Li

**Affiliations:** 1 Department of Obstetrics, Gynecology, and Reproductive Science, Icahn School of Medicine at Mount Sinai, New York, NY USA; 2 Sema4, Stamford, CT USA; 3 Department of Pediatrics, Icahn School of Medicine at Mount Sinai, New York, NY USA; 4 Department of Genetics and Genomic Sciences, The Icahn Institute for Genomics and Multiscale Biology, Icahn School of Medicine at Mount Sinai, New York, NY USA

**Keywords:** Late preterm delivery, Neonatal respiratory outcomes, Betamethasone, Dexamethasone, Hypoglycemia, Late preterm infant, Late preterm twin, Respiratory distress syndrome

## Abstract

**Background:**

In 2016, the American College of Obstetricians and Gynecologists recommended antenatal corticosteroids in the late preterm period for women at risk for preterm delivery. Limited real-world evidence exists on neonatal outcomes, particularly for twin gestations, following the guideline change. The study objective is to determine the association of antenatal corticosteroids in late preterm singleton and twin pregnancies with respiratory complications and hypoglycemia in a real-world clinical setting.

**Methods:**

This is a retrospective cohort study comprising late preterm deliveries (4,341 mother–child pairs) within the Mount Sinai Health System, 2012–2018. The exposure of interest is antenatal corticosteroid administration of betamethasone during pregnancy between 34 0/7 and 36 6/7 weeks. Our primary outcomes are neonatal respiratory complications and hypoglycemia. Multivariable logistic regression was used to estimate the association between antenatal corticosteroid exposure and these two outcomes. We stratified the study population by singleton gestations and twins to minimize the potential confounding from different obstetric management between the two groups.

**Results:**

Among a total of 4,341 mother–child pairs (3,309 *singleton* and 1,032 *twin* mother–child pairs), 745 mothers received betamethasone, of which 40.94% (305/745) received the full course. Relative to no treatment, a full course of betamethasone was associated with reduced odds of respiratory complications (OR = 0.53, 95% CI:[0.31–0.85], p < 0.01) and increased odds of hypoglycemia (OR = 1.86, 95%CI:[1.34–2.56], p < 0.01) in singletons; however, the association with respiratory complications was not significant in twins (OR = 0.42, 95% CI:[0.11–1.23], *p* = 0.16), but was associated with increased odds of hypoglycemia (OR = 2.18, 95% CI:[1.12–4.10], *p* = 0.02).

A partial course of betamethasone (relative to no treatment) was not significantly associated with any of the outcomes, other than respiratory complications in twins (OR = 0.34, 95% CI:[0.12–0.82], *p* = 0.02).

**Conclusions:**

Exposure to antenatal corticosteroids in singletons and twins is associated with increased odds of hypoglycemia. Among singletons, exposure to the full dosage (i.e. two doses) was associated with decreased odds of respiratory complications but this was only the case for partial dose among twins. Twin gestations were not studied by the Antenatal Late Preterm Steroids trial. Therefore, our study findings will contribute to the paucity of evidence on the benefit of antenatal corticosteroids in this group. Health systems should systematically monitor guideline implementations to improve patient outcomes.

**Supplementary Information:**

The online version contains supplementary material available at 10.1186/s12884-022-05262-1.

## Introduction

Antenatal corticosteroid (ACS) use has been widely supported in the United States (US) in pregnancies at risk for early preterm delivery (< 34 gestational weeks) to accelerate fetal lung maturation [1]. Benefits include reduced neonatal morbidity such as respiratory distress syndrome, intraventricular hemorrhage, neonatal intensive care unit (NICU) admission, as well as death [1, 2]. The Antenatal Late Preterm Steroids (ALPS) trial published in 2016 demonstrated that betamethasone use in singleton gestations in late preterm pregnancies (34–36 gestational weeks) at high risk for preterm delivery, significantly reduced the rate of neonatal respiratory complications, but increased the rate of neonatal hypoglycemia [3]. Consequently, since 2016, the American College of Obstetricians and Gynecologists (ACOG) and the Society for Maternal–Fetal Medicine have recommended ACS for women with a singleton pregnancy between 34 0/7 and 36 6/7 weeks at imminent risk of preterm birth within 7 days [4–6].

Obstetric practices, including those at our own institution, increasingly administered betamethasone for women at risk of late preterm delivery, within and outside of the criteria specified in the ALPS trial [7–9]. Though administration of ACS for twins and other deviations have been documented, the benefits remain unclear [9, 10]. Twins and singletons may have biological and pharmacokinetic differences [11], so findings from the ALPS trial may not be generalizable to twin gestations [12, 13]. Neonatal hypoglycemia has also been linked to developmental delays [14, 15], but the relationship is not conclusive [16]. Given that approximately 70% of preterm births occur at 34–36 weeks’ gestation representing 7.5% of all births [17], the implementation of late preterm ACS has potentially far-reaching consequences with uncertain long-term benefits, underscoring the importance of demonstrating real-world effects.

It remains unclear whether the administration of ACS in twin gestations confer the same benefits of reduced respiratory morbidity as well as effects of hypoglycemia as seen in singletons. To date, there is limited evidence documenting the benefits of late preterm steroids in twin gestations [18]. Thus, our study aimed to provide real-world evidence to assess the effect of betamethasone, accounting for dose and timing, in late preterm singleton and twin gestations on neonatal respiratory complications and hypoglycemia.

## Materials and Methods

### Study population

**Fig. 1:**
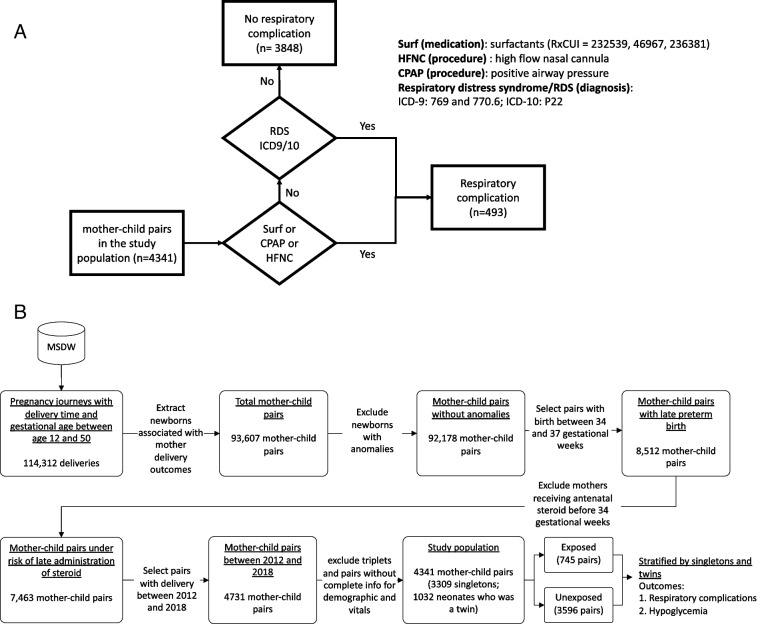
**a** Respiratory Complications Phenotyping in Study Population, **b** Selection Flow of Mother–Child Pairs

We used deidentified Electronic Medical Record (EMR) data from the Mount Sinai Health System (MSHS) [19]. Patients with late preterm birth (between 34 0/7 and 36 6/7 weeks of pregnancy) who had never received ACS before 34 weeks of the current pregnancy between 2012 and 2018 were considered eligible for the study. Delivery time and the corresponding gestational age were harmonized using standardized delivery summaries completed by the Labor & Delivery department staff at MSHS. We excluded patients who: i) did not have admission time for delivery or gestational age, ii) were pregnant with triplets or higher multiples, iii) did not have complete demographic information, iv) were newborns with the following (serving as a proxy to potential congenital anomalies): postnatal genetic diagnostic tests, metabolic diagnostic tests, and heart surgeries. The inclusion and exclusion criteria are presented in Fig. 1. The study was approved by the Institutional Review Board at the Icahn School of Medicine at Mount Sinai (IRB-21–00,824). Informed consent from patient is not required as the study uses de-identified data under the HIPAA privacy rule.

### Exposure

Exposure in the study is defined as receiving at least one dose of ACS between 34 0/0 and 36 6/7 weeks for the first time in the current pregnancy. The protocol for ACS administration at MSHS is a course of two intramuscular injections consisting of 12 mg of betamethasone given 24 h apart. We identified patients who received steroid treatment, as well as the timing and number of doses, using information from RxNorm, a naming system for all medications available in the US market maintained by the US National Library of Medicine (we used the concept unique identifier, RxCUI = 1514, for betamethasone) [20], administration methods, and dosage in the medication records. Receiving at least one dose of ACS was studied to evaluate its overall effectiveness. To assess the dose effect of ACS, we considered whether patients were: i) exposed to the *full course* of treatment (*two doses*), ii) exposed only to a *partial course* (*one dose*), or iii) never exposed to ACS. We calculated the number of days from receiving the first dose to delivery by coding the value for patients who never received ACS as 0, who received within 24 h prior to delivery as 1, who received 24-48 h prior to delivery as 2 etc., to evaluate the effect of the timing of exposure to ACS.

### Outcomes

Our primary outcomes of interest are neonatal respiratory complications and hypoglycemia.

i) Newborns were considered as having respiratory complications if, within 72 h after delivery, they were diagnosed with either respiratory distress syndrome or received treatment for respiratory symptoms, i.e., continuous positive airway pressure, high-flow nasal cannula, and surfactants (Fig. 1a).
ii) Newborns with glucose levels < 40 mg/dL on ≥ 2 occasions within 72 h of birth were considered as having hypoglycemia.

### Covariates

Patients’ demographic variables – race and ethnicity, age at delivery, and insurance – were obtained through the EMR. We calculated maternal body mass index (BMI) as weight(kg)/height [2](m [2]) at delivery. Length of pregnancy by week (i.e. gestational age), and mode of delivery were identified using delivery summaries, and ICD9/10 and procedure codes as described in our previous study [21]. Antepartum preeclampsia during pregnancy was determined using electronic phenotyping [22]. Patients receiving medication for gestational diabetes mellitus (insulin, metformin, and glyburide) were included. Birth year (i.e. delivery year), birth weight, sex of newborns, and 5-min Apgar score (using 1-min Apgar score if the 5-min was not available) were also included in the analyses.

### Statistical analysis

We used multivariable logistic regression to estimate the association between ACS exposure and respiratory complications and hypoglycemia. In the analysis of respiratory complications and hypoglycemia, we stratified the study population by singleton gestations and twins to minimize the potential confounding from different obstetric management between the two groups. For all the multivariable models, we adjusted for: maternal race/ethnicity, age at delivery, insurance status, BMI at delivery, gestational age at delivery, mode of delivery, antepartum preeclampsia, medication for gestational diabetes, as well as the newborns’ birth year (continuous), birth weight (continuous), sex, and Apgar score (continuous). Statistical analyses were performed using R version 4.0.5 [23].

## Results

### Characteristics of the study population

**Fig. 2:**
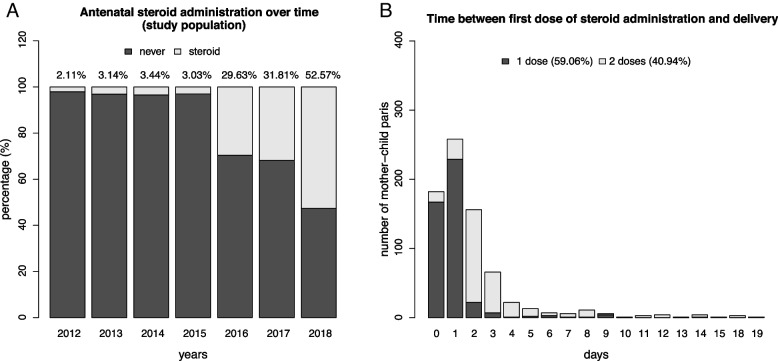
**a** Antenatal Corticosteroid (Betamethasone) Administration in Study Population, 2012–2018, **b** Days Between Receiving First Betamethasone Dose to Delivery

Out of 114,312 deliveries identified in MSHS, 4,341 mother–child pairs between 2012 and 2018 were eligible for the study (Fig. 1b). Among the eligible mother–child pairs, there were 3,309 (76.23%) mother–child pairs in singletons and 1,032 (23.77%) mother–child pairs in twins (Fig. 1b). The prevalence of receiving ACS increased over time especially after 2016 (from 2.11% in 2012 to 52.57% in 2018) (Fig. 2a). Of the 745 mother–child pairs who received ACS in the study (631 singletons; 114 twins), only 40.94% completed the full course and 59.06% received one dose within one day of delivery, and none received more than two doses (Fig. 2b, Supplementary Table 1). Characteristics of the study population by betamethasone exposure are summarized in Table 1. Compared to neonates whose mother did not receive ACS, those who had received ACS had a significantly higher percentage of hypoglycemia (20.7% vs. 17.3%, *p* = 0.03). Proportions of respiratory complications between the two groups were similar. Apart from preeclampsia, maternal BMI at delivery, delivery mode, newborn’s sex, and time from birth to the last record in MSHS, the distributions of most of the covariates were statistically different between the groups with/without receiving antenatal steroid (p < 0.05, Table 1). Descriptive characteristics by outcome status are in Supplementary Tables 2a-b.

**Table 1:** Descriptive Statistics According to Antenatal Corticosteroid (Betamethasone) Exposure, Stratified by Singleton and Twin Gestations^a^

	Total mother–child pairs			Singleton pairs			Twin pairs		
	Unexposed group	Betamethasone group	*p*	Unexposed group	Betamethasone group	*p*	Unexposed group	Betamethasone group	*p*
*n*	3596	745		2678	631		918	114	
**Neonatal outcomes**									
Respiratory complications (%)	395 (11.0)	98 (13.2)	0.102	281 (10.5)	87 (13.8)	0.022	114 (12.4)	11 (9.6)	0.482
Hypoglycemia (%)	621 (17.3)	154 (20.7)	0.031	444 (16.6)	130 (20.6)	0.019	177 (19.3)	24 (21.1)	0.745
**Maternal Characteristics**									
Age at delivery (mean (SD))	33.18 (6.16)	32.63 (6.11)	0.029	32.67 (6.08)	32.55 (6.13)	0.655	34.65 (6.15)	33.11 (6.03)	0.011
Race/ethnicity (%)			< 0.001			< 0.001			< 0.001
African-American	413 (11.5)	121 (16.2)		345 (12.9)	101 (16.0)		68 (7.4)	20 (17.5)	
Asian	261 (7.3)	53 (7.1)		187 (7.0)	45 (7.1)		74 (8.1)	8 (7.0)	
Caucasian/White	2089 (58.1)	339 (45.5)		1451 (54.2)	279 (44.2)		638 (69.5)	60 (52.6)	
Hispanic/Latino	558 (15.5)	160 (21.5)		484 (18.1)	144 (22.8)		74 (8.1)	16 (14.0)	
Other	231 (6.4)	60 (8.1)		187 (7.0)	50 (7.9)		44 (4.8)	10 (8.8)	
Unknown	44 (1.2)	12 (1.6)		24 (0.9)	12 (1.9)		20 (2.2)	0 (0.0)	
Private insurance (%)	2570 (71.5)	458 (61.5)	< 0.001	1770 (66.1)	380 (60.2)	0.006	800 (87.1)	78 (68.4)	< 0.001
BMI at delivery (mean (SD))	29.99 (5.90)	30.14 (5.94)	0.546	29.84 (6.21)	30.01 (6.07)	0.535	30.45 (4.85)	30.85 (5.07)	0.403
Gestational age (weeks) (mean (SD))	36.55 (0.68)	36.31 (0.72)	< 0.001	36.63 (0.63)	36.35 (0.70)	< 0.001	36.34 (0.77)	36.05 (0.79)	< 0.001
Cesarean delivery (%)	2031 (56.5)	439 (58.9)	0.235	1261 (47.1)	345 (54.7)	0.001	770 (83.9)	94 (82.5)	0.8
Preeclampsia (%)	169 (4.7)	31 (4.2)	0.588	123 (4.6)	23 (3.6)	0.35	46 (5.0)	8 (7.0)	0.494
GDM medication (%)	733 (20.4)	185 (24.8)	0.008	639 (23.9)	163 (25.8)	0.323	94 (10.2)	22 (19.3)	0.006
Gestational Weeks (%)			< 0.001			< 0.001			< 0.001
34	393 (10.9)	116 (15.6)		221 (8.3)	84 (13.3)		172 (18.7)	32 (28.1)	
35	817 (22.7)	284 (38.1)		555 (20.7)	240 (38.0)		262 (28.5)	44 (38.6)	
36	2386 (66.4)	345 (46.3)		1902 (71.0)	307 (48.7)		484 (52.7)	38 (33.3)	
**Neonate covariates**									
Birth weight (mean (SD))	2.57 (0.43)	2.49 (0.40)	< 0.001	2.67 (0.41)	2.54 (0.40)	< 0.001	2.27 (0.33)	2.22 (0.30)	0.133
^b^Birth weight below 10th percentile	304 (8.5)	97 (13.0)	< 0.001	143 (5.3)	70 (11.1)	< 0.001	161 (17.5)	27 (23.7)	0.14
^c^Birth weight below 3^rd^ percentile	132 (3.7)	21 (2.8)	0.29	53 (2.0)	12 (1.9)	1.00	79 (8.6)	9 (7.9)	0.937
Newborn sex = Male (%)	1903 (52.9)	396 (53.2)	0.939	1426 (53.2)	339 (53.7)	0.864	477 (52.0)	57 (50.0)	0.767
Private insurance (%)	2488 (69.2)	434 (58.3)	< 0.001	1714 (64.0)	360 (57.1)	0.001	774 (84.3)	74 (64.9)	< 0.001
Apgar score (mean (SD))	8.94 (0.34)	8.88 (0.48)	< 0.001	8.94 (0.36)	8.88 (0.48)	0.001	8.94 (0.29)	8.88 (0.42)	0.045
Apgar < 7 (%)	13 (0.4)	6 (0.8)	0.172	13 (0.5)	5 (0.8)	0.521	0 (0.0)	1 (0.9)	0.214
NICU admission (%)	455 (12.7)	148 (19.9)	< 0.001	302 (11.3)	122 (19.3)	< 0.001	153 (16.7)	26 (22.8)	0.133
Year of delivery (%)			< 0.001			< 0.001			< 0.001
2012	556 (15.5)	12 (1.6)		410 (15.3)	8 (1.3)		146 (15.9)	4 (3.5)	
2013	587 (16.3)	19 (2.6)		427 (15.9)	15 (2.4)		160 (17.4)	4 (3.5)	
2014	618 (17.2)	22 (3.0)		460 (17.2)	16 (2.5)		158 (17.2)	6 (5.3)	
2015	640 (17.8)	20 (2.7)		478 (17.8)	20 (3.2)		162 (17.6)	0 (0.0)	
2016	475 (13.2)	200 (26.8)		351 (13.1)	164 (26.0)		124 (13.5)	36 (31.6)	
2017	508 (14.1)	237 (31.8)		406 (15.2)	199 (31.5)		102 (11.1)	38 (33.3)	
2018	212 (5.9)	235 (31.5)		146 (5.5)	209 (33.1)		66 (7.2)	26 (22.8)	

^a^We summarized the categorical variables with counts (percentage) and the continuous variables with mean (standard deviation (SD)) and compared the difference of covariates between groups either by exposure status or outcome status using chi-square test for categorical variables and t-test for continuous variables

^b,c^Proportions were calculated based on a 2017 US Reference for Birth Weight Percentiles by Aris et al. 2019 [37]

### Receiving antenatal steroids is associated with reduced odds of neonatal respiratory complications

**Fig. 3:**
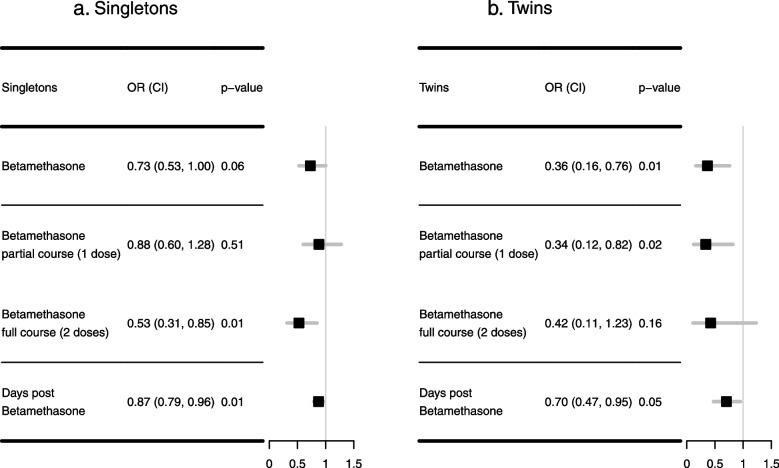
Association Between Antenatal Corticosteroid (Betamethasone) and Neonatal Respiratory Complications: **a** shows the results in singletons and **b** shows the result in twins. Both tables include results of 1) any use of betamethasone, 2) full course (2 doses) of betamethasone vs. no betamethasone, 3) partial course (1 doses) of betamethasone vs. no betamethasone, and 4) days from the first dose of betamethasone to delivery

Multivariable regressions for neonatal respiratory complications are summarized in Fig. 3a-b. We found that any betamethasone use (i.e., at least one dose), had no significant association with respiratory complications in singletons (OR = 0.73, 95% CI:[0.53–1.00], *p* = 0.06) compared to no betamethasone use, while there was a 64% decrease in odds of respiratory complications in twins (OR = 0.36, 95% CI:[0.16–0.76], *p* = 0.01).

Additionally, we assessed whether there was an ACS dose effect (i.e., zero vs. one vs. two doses) (Fig. 3a-b). In singletons, those receiving two doses of betamethasone were associated with reduced odds of respiratory complications as compared to those not receiving any, (OR = 0.53, 95% CI:[0.31–0.85], *p* = 0.01); however, those receiving one dose did not show a significant difference in respiratory complications (OR = 0.88, 95% CI:[0.60–1.28], *p* = 0.51). Compared to twins not exposed to any ACS, there was no significant difference in respiratory complications in twins receiving two doses of betamethasone (OR = 0.42, 95% CI:[0.11–1.23], *p* = 0.16); however, receiving one dose was associated with reduced odds of respiratory complications (OR = 0.34, 95% CI:[0.12–0.82], *p* = 0.02).

We also assessed the association of the timing of ACS administration and respiratory complications. Newborns had lower odds of developing respiratory complications if their mothers received betamethasone earlier in both singletons (OR = 0.87, 95% CI:[0.79–0.96], *p* = 0.01) and twins (OR = 0.70, 95% CI:[0.47–0.95], *p* = 0.05) (Fig. 3a-b). The full regression model outputs are in Supplementary Figs. 1–2.

### Receiving antenatal steroids is associated with increased odds of neonatal hypoglycemia

**Fig. 4:**
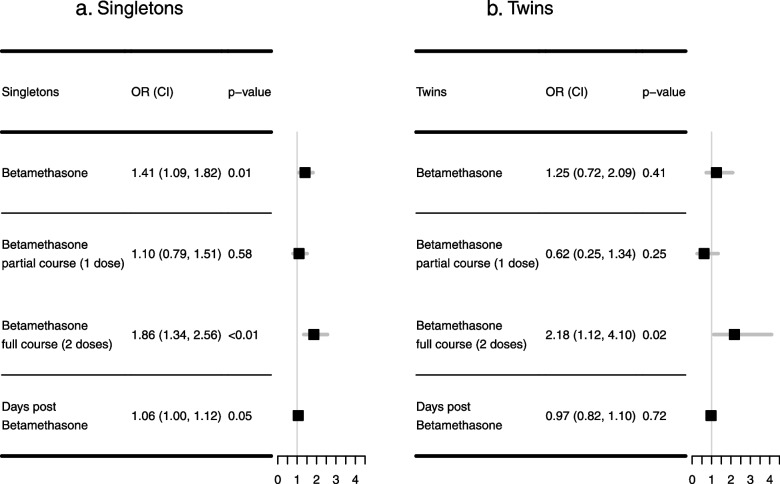
Association Between Antenatal Corticosteroid (Betamethasone) and Neonatal Hypoglycemia: **a** shows the results in singletons and **b** shows the result in twins. Both tables include results of 1) any use of betamethasone, 2) full course (2 doses) of betamethasone vs. no betamethasone, 3) partial course (1 doses) of betamethasone vs. no betamethasone, and 4) days from the first dose of betamethasone to delivery

In singletons, across all dosing groups betamethasone exposure was associated with increased odds of developing hypoglycemia within 72 h of birth (OR = 1.41, 95% CI:[1.09–1.82], *p* = 0.01, Fig. 4a). Relative to the group not receiving betamethasone, there was no significant association between hypoglycemia and receiving one dose of betamethasone (OR = 1.10, 95% CI:[0.79–1.51], *p* = 0.58) (Fig. 4a), although receiving two doses was associated with increased odds (OR = 1.86, 95% CI:[1.34–2.56], *p* < 0.01) of hypoglycemia.

In twins, being exposed to at least one dose of betamethasone was not significantly associated with hypoglycemia (OR = 1.25, 95% CI:[0.72–2.09], *p* = 0.41, Fig. 4b). However, receiving two doses was associated with increased odds of having hypoglycemia compared to no betamethasone (OR = 2.18, 95% CI:[1.12–4.10], *p* = 0.02, Fig. 4b). The association was not statistically significant in twins receiving one dose of betamethasone relative to no doses (OR = 0.62, 95% CI:[0.25–1.34], *p* = 0.25).

In addition, longer intervals between the first betamethasone administration and delivery were associated with increased odds of hypoglycemia in singletons (OR = 1.06, 95% CI:[1.00–1.12], *p* = 0.05), but the association in twins was not statistically significant (OR = 0.97, 95% CI:[0.82–1.10], *p* = 0.72, Fig. 4a-b).

## Discussion

### Principal Findings

In this late preterm cohort, the use of ACS has been increasing since 2016, in response to ACOG guidelines (Fig. 2a). Patients receiving a full course of ACS i) were less likely to have neonatal respiratory complications but more likely to have hypoglycemia among singletons; ii) had increased odds of hypoglycemia but no significant association with respiratory complications among twins. Although a partial course was associated with reduced respiratory complications among twins, the association with other outcomes was not conclusive.

### Results in the Context of What is Known

Among singletons receiving a full course of ACS, our result of decreased respiratory complications is consistent with the ALPS trial, as well as retrospective observational studies [3, 24, 25]. Still, others have found no reduction [26–29]. It has been suggested that the distribution of pregnancies by gestational age could explain the different findings between studies as ACS may be more effective in reducing respiratory morbidity due to lung immaturity at earlier gestational ages within the late preterm period [25]. Our study includes a gestational age distribution similar to previous studies with adequate representation of both earlier and later gestational ages between 34 and 36 weeks [25, 30, 31]. In terms of hypoglycemia, our findings were largely consistent with what has been found [28, 30–32]. Our definition of hypoglycemia was compatible with the ALPS trial. However, different cutoffs in blood glucose levels (e.g. 50 mg/dl [28]) and time after delivery (e.g. within 48 hr [32].) have been reported, making direct comparisons difficult. For twin gestations that received the full course of ACS, our finding is consistent with a study in Israel [12]. However, the gestational age at administration might be more important than ACS exposure in twins [12, 13, 33].

We examined partial dosing and days of ACS administration from delivery. Results indicate that exposure to partial dosing of betamethasone among singletons was not associated with reduced odds of respiratory complications, in contrast to those receiving the full course. Among twins, we found the opposite; the reason for this is unclear but there are potential physiologic and metabolic differences between twin and singleton gestations [11]. Receiving only a partial course of betamethasone compared to none did not increase the odds of hypoglycemia among either singletons or twins, suggestive of a potential dose–effect. While Janssen et al. evaluated number of doses and saw no significant associations with respiratory complications, the variable entered in their model was continuous, making a direct comparison difficult. [25]

Regarding ACS-to-delivery interval, few have examined this; we showed that longer intervals were associated with decreased odds of respiratory complications in both singleton and twin gestations. A retrospective cohort study in Korea in early preterm twins reported that an interval of 2–7 days was associated with decreased odds of respiratory distress syndrome, but not with an interval of fewer than 2 days, when compared to those unexposed to ACS [34]. However, in Janssen et al., reduced odds of respiratory complications was only shown when ACS was administered within 2 days, but not within 7 days of delivery among late preterm newborns. [25] A recent study in the US has shown that newborns who have been exposed to ACS were more likely to have severe hypoglycemia within a one-day interval when compared to unexposed newborns [35].

### Strengths and Limitations

The major strength of this study is that it includes a large study cohort with diversity in provider types allowing for the opportunity to evaluate the implementation of ACOG guidelines outside research settings. With “any betamethasone use” as an exposure, we provide an estimation of the overall effectiveness of the new ACOG guidelines in MSHS. We also examined the effects of deviations from what has been studied in the ALPS trial including twin gestations and partial dosing of ACS, which is commonly seen in clinical settings, yet lacks definitive clinical evidence. As we await an ongoing trial evaluating ACS in late preterm twin gestations [18], our study adds to the nascent evidence base for this group. Our study also included patients taking gestational diabetes medication, unlike others [26–28]. Until more definitive evidence is available for groups not studied in the ALPS trial, this study supports shared decision-making between patient and provider in discussing potential benefits and unknown long-term outcomes.

We acknowledge several limitations of our study. It is retrospective and subject to inherent ascertainment bias and unmeasured confounding. Additionally, we did not account for glucose levels after 72 h of birth and postnatal management in the study; therefore, we could not distinguish between transient and persistent hypoglycemia. Future research should address this limitation as only ~ 2% in the ALPS cohort had persistent hypoglycemia [36]. We may not have a large enough sample to detect significant associations among the twins exposed to full course of ACS. The structured EMR data used in this study lacked granularity to assess for provider practice differences, length of respiratory support, and adverse events related to hypoglycemia. We also acknowledge that since ICD codes are primarily used for billing purposes, we may not have captured the full relevant cohort of newborns.

## Conclusions

ACS administration adherent to ACOG guidelines reflected the findings of the ALPS trial, and was similar for twin gestations. We believe that the study findings based on real-world implementation of clinical guidelines are critical to informing the effect of ACS outside research settings, and health systems should systematically monitor how guidelines are implemented to improve the health of patients.

## Supplementary Information

**Additional file 1.**

## Data Availability

The clinical data here were used under license from Mount Sinai Data Warehouse in the current study. As a result, this dataset is not publicly available. Qualified researchers affiliated with the Mount Sinai Health Systems may apply for access to these data through the Mount Sinai Health Systems Institutional Review Board. We used R version 4.0.5 to analyze our data, and will release the code under the CC BY-NC-SA 3.0 license (https://creativecommons.org/licenses/by-nc-sa/3.0/).

## List of abbreviations

ACOG: American College of Obstetricians and Gynecologists
ACS: Antenatal corticosteroid
ALPS: Antenatal Late Preterm Steroids
BMI: Body mass index
EMR: Electronic medical record
MSHS: Mount Sinai Health System
NICU: Neonatal intensive care unit
