# Supplementary material for: Late preterm antenatal corticosteroids in singleton and twin gestations: a retrospective cohort study
Source: BMC Pregnancy Childbirth. 2022 Dec 5;22:904. doi: 10.1186/s12884-022-05262-1 (PMC9721054; doi:10.1186/s12884-022-05262-1)
Supplement: Supplementary file 1 — Additional file 1. [file 12884_2022_5262_MOESM1_ESM.docx]

**Title: Late preterm antenatal corticosteroid and health outcomes of newborns: A retrospective cohort study**

Supplementary Material

Supplementary Table 1: Number of Betamethasone Doses Received by Singleton vs Twin Gestations

|  | n (%) | |  |
| --- | --- | --- | --- |
|  | 1 dose | 2 doses | Total |
| Singletons | 380 (60.22%) | 251 (38.78%) | 631 |
| Twins | 60 (52.63%) | 54 (47.37%) | 114 |
| Total | 440 | 305 |  |

Supplementary Table 2a Mother and Neonate Characteristics by Outcome Status: Respiratory Complications

|  | Group without respiratory complications | Group with respiratory complications | p |
| --- | --- | --- | --- |
| n | 3848 | 493 |  |
|  |  |  |  |
| **Mother covariates** |  |  |  |
| Age at delivery (mean (SD)) | 33.05 (6.14) | 33.33 (6.22) | 0.336 |
| Race/ethnicity (%) |  |  | 0.042 |
| African-American | 477 (12.4) | 57 (11.6) |  |
| Asian | 290 (7.5) | 24 (4.9) |  |
| Caucasian/White | 2124 (55.2) | 304 (61.7) |  |
| Hispanic/Latino | 640 (16.6) | 78 (15.8) |  |
| Other | 268 (7.0) | 23 (4.7) |  |
| Unknown | 49 (1.3) | 7 (1.4) |  |
| Private insurance (%) | 2673 (69.5) | 355 (72.0) | 0.269 |
| BMI at delivery (mean (SD)) | 30.01 (5.90) | 30.08 (5.94) | 0.797 |
| Gestational age (weeks) (mean (SD)) | 36.57 (0.65) | 36.05 (0.84) | <0.001 |
| Cesarean delivery (%) | 2135 (55.5) | 335 (68.0) | <0.001 |
| Preeclampsia (%) | 183 (4.8) | 17 (3.4) | 0.234 |
| GDM medication (%) | 831 (21.6) | 87 (17.6) | 0.05 |
|  |  |  |  |
| **Neonate covariates** |  |  |  |
| Birth weight (mean (SD)) | 2.57 (0.42) | 2.40 (0.46) | <0.001 |
| Newborn sex= Male (%) | 2000 (52.0) | 299 (60.6) | <0.001 |
| Private insurance (%) | 2590 (67.3) | 332 (67.3) | >0.99 |
| Apgar score (mean (SD)) | 8.96 (0.27) | 8.69 (0.75) | <0.001 |
| NICU admission (%) | 309 (8.0) | 294 (59.6) | <0.001 |
| Year of delivery (%) |  |  | <0.001 |
| 2012 | 518 (13.5) | 50 (10.1) |  |
| 2013 | 554 (14.4) | 52 (10.5) |  |
| 2014 | 555 (14.4) | 85 (17.2) |  |
| 2015 | 585 (15.2) | 75 (15.2) |  |
| 2016 | 612 (15.9) | 63 (12.8) |  |
| 2017 | 653 (17.0) | 92 (18.7) |  |
| 2018 | 371 (9.6) | 76 (15.4) |  |
| Days from birth to the last record in MSHS (mean (SD)) | 316.75 (410.19) | 305.08 (386.11) | 0.55 |

Supplementary Table 2b Mother and Neonate Characteristics by Outcome Status: Hypoglycemia

|  | Group without hypoglycemia | Group with hypoglycemia | p |
| --- | --- | --- | --- |
| n | 3566 | 775 |  |
|  |  |  |  |
| **Mother covariates** |  |  |  |
| Age at delivery (mean (SD)) | 32.99 (6.19) | 33.53 (5.98) | 0.025 |
| Race/ethnicity (%) |  |  | <0.001 |
| African-American | 463 (13.0) | 71 (9.2) |  |
| Asian | 271 (7.6) | 43 (5.5) |  |
| Caucasian/White | 1935 (54.3) | 493 (63.6) |  |
| Hispanic/Latino | 610 (17.1) | 108 (13.9) |  |
| Other | 240 (6.7) | 51 (6.6) |  |
| Unknown | 47 (1.3) | 9 (1.2) |  |
| Private insurance (%) | 2457 (68.9) | 571 (73.7) | 0.01 |
| BMI at delivery (mean (SD)) | 30.10 (5.98) | 29.64 (5.52) | 0.047 |
| Gestational age (weeks) (mean (SD)) | 36.54 (0.69) | 36.38 (0.69) | <0.001 |
| Cesarean delivery (%) | 2002 (56.1) | 468 (60.4) | 0.034 |
| Preeclampsia (%) | 173 (4.9) | 27 (3.5) | 0.121 |
| GDM medication (%) | 766 (21.5) | 152 (19.6) | 0.269 |
|  |  |  |  |
| **Neonate covariates** |  |  |  |
| Birth weight (mean (SD)) | 2.56 (0.42) | 2.54 (0.44) | 0.24 |
| Newborn sex = Male (%) | 1863 (52.2) | 436 (56.3) | 0.047 |
| Private insurance (%) | 2370 (66.5) | 552 (71.2) | 0.012 |
| Apgar score (mean (SD)) | 8.93 (0.37) | 8.93 (0.35) | 0.533 |
| NICU admission (%) | 504 (14.1) | 99 (12.8) | 0.35 |
| Year of delivery (%) |  |  | <0.001 |
| 2012 | 484 (13.6) | 84 (10.8) |  |
| 2013 | 491 (13.8) | 115 (14.8) |  |
| 2014 | 524 (14.7) | 116 (15.0) |  |
| 2015 | 524 (14.7) | 136 (17.5) |  |
| 2016 | 505 (14.2) | 170 (21.9) |  |
| 2017 | 607 (17.0) | 138 (17.8) |  |
| 2018 | 431 (12.1) | 16 (2.1) |  |
| Days from birth to the last record in MSHS (mean (SD)) | 317.91 (407.46) | 303.96 (407.76) | 0.388 |

Supplementary Figure 1a: Association Between Any Antenatal Corticosteroid (Betamethasone) Use and Covariates on Respiratory Complications in Singleton Births

Supplementary Figure 1b: Association Between Any Antenatal Corticosteroid (Betamethasone) Use and Covariates on Respiratory Complications in Twin Births

Supplementary Figure 1c: Dose-effect of Antenatal Corticosteroid (Betamethasone) Use on Respiratory Complications in Singleton Births

Supplementary Figure 1d: Dose-effect of Antenatal Corticosteroid (Betamethasone) Use on Respiratory Complications in Twin Births

Supplementary Figure 1e: Association between *Days from First Dose of Betamethasone to Delivery* and Respiratory Complications in Singleton Births

Supplementary Figure 1f: Association between *Days from First Dose of Betamethasone to Delivery* and Respiratory Complications in Twin Births

Supplementary Figure 2a: Association between Any Antenatal Corticosteroid (Betamethasone) Use and Covariates on Hypoglycemia in Singleton Births

Supplementary Figure 2b: Association between Any Antenatal Corticosteroid (Betamethasone) Use and Covariates on Hypoglycemia in Twin Births

Supplementary Figure 2c: Dose-effect of Antenatal Corticosteroid (Betamethasone) Use on Hypoglycemia in Singleton Births

Supplementary Figure 2d: Dose-effect of Antenatal Corticosteroid (Betamethasone) Use on Hypoglycemia in Twin Births

Supplementary Figure 2e: Association between *Days from First Dose of Betamethasone to Delivery* and Hypoglycemia in Singleton Births

Supplementary Figure 2f: Association between *Days from First Dose of Betamethasone to Delivery* and Hypoglycemia in Twin Births
